# Punch Biopsy as a Diagnostic Keystone for Metastatic Cardiac Angiosarcoma Treated With Anthracycline-Based Chemotherapy: A Case Report

**DOI:** 10.7759/cureus.79524

**Published:** 2025-02-23

**Authors:** Aonghus Joyce, Gráinne Murphy, Cynthia C Heffron, David Aherne, Richard M Bambury

**Affiliations:** 1 Medical Oncology, Cork University Hospital, Cork, IRL; 2 Rheumatology, Cork University Hospital, Cork, IRL; 3 Histopathology, Cork University Hospital, Cork, IRL; 4 Cardiothoracic Surgery, Cork University Hospital, Cork, IRL

**Keywords:** cardiac angiosarcoma, cutaneous dermal metastasis, kras mutation, rare cancers, rare case report

## Abstract

Angiosarcoma is a rare, aggressive malignancy originating from endothelial cells and associated with a poor prognosis. Diagnosis is histologically challenging. We present a rare case of cardiac angiosarcoma involving multiple presentations and investigations, which were inconclusive until a diagnosis was finally reached on a biopsy of a cutaneous lesion. Clinical and radiologic response to systemic anthracycline-based chemotherapy is discussed. This is the first reported case of right atrial primary cardiac angiosarcoma presenting with cardiac symptoms rather than systemic symptoms. The case illustrates the challenges associated with the diagnosis of this rare malignancy. It also highlights the feasibility of using anthracycline-based chemotherapy despite the potential cardiotoxicity of these agents.

## Introduction

Primary cardiac neoplasms are extremely rare, with an incidence of 1.4 per 100,000 persons per year [1]. One-quarter of these are malignant. Angiosarcoma is a rare, aggressive malignancy originating from endothelial cells [2]. Sixty percent of angiosarcomas originate in the head and neck, with rarer sites of origin including the liver, breast, and spleen. Angiosarcomas represent 2% of all soft tissue sarcomas and 33% of all primary malignant cardiac neoplasms, representing the most common histological subtype. Diagnosis is histologically challenging [3]. The prognosis for angiosarcoma is poor, with a 60% five-year overall survival rate in localized disease and a median overall survival of 3-10 months for metastatic disease [4]. Prognostic indicators associated with poor overall survival for patients with metastatic disease include the presence of liver metastases and a higher Eastern Cooperative Oncology Group Performance Status (ECOG PS) score [3]. We present a case of this malignancy involving multiple presentations and investigations, which were inconclusive until angiosarcoma was diagnosed on biopsy of a metastatic cutaneous lesion. Clinical and radiologic response to systemic chemotherapy is discussed.

This case report was previously presented as a meeting abstract at the Irish Society for Medical Oncology Bursary Awards Meeting on January 27, 2023. The case report was also previously presented as a poster at the Royal College of Physicians of Ireland Trainees' Committee Awards on May 26, 2023, and at the Cork University Hospital Quality Improvement, Audit & Research Symposium on June 21, 2024.

## Case presentation

A 49-year-old man initially presented to another medical institution with chest pain. Pericarditis was diagnosed at that time, and the patient commenced colchicine and non-steroidal anti-inflammatory drugs (NSAIDs). There was no medical or family history of note. The patient did have an extensive travel history, including recent trips to Southeast Asia and North America. Two months later, he was admitted to our institution with collapse secondary to pericarditis, cardiac tamponade, and a left-sided pleural effusion. At that time, an emergency pericardiocentesis was performed. Computed tomography (CT) thorax demonstrated pleural and pericardial effusions but was otherwise unremarkable. Pleural fluid cytology from a thoracocentesis procedure was benign. The patient's symptoms responded to high-dose corticosteroids. Extensive autoimmune, infectious, and auto-inflammatory screening was conducted. A diagnosis of autoimmune pericarditis was reached, although autoimmune serology was negative. Anakinra was commenced during outpatient follow-up. Six months later, he presented to our institution with frank hemoptysis, malaise, and proximal limb myalgia.

Investigations

**Figure 1:**
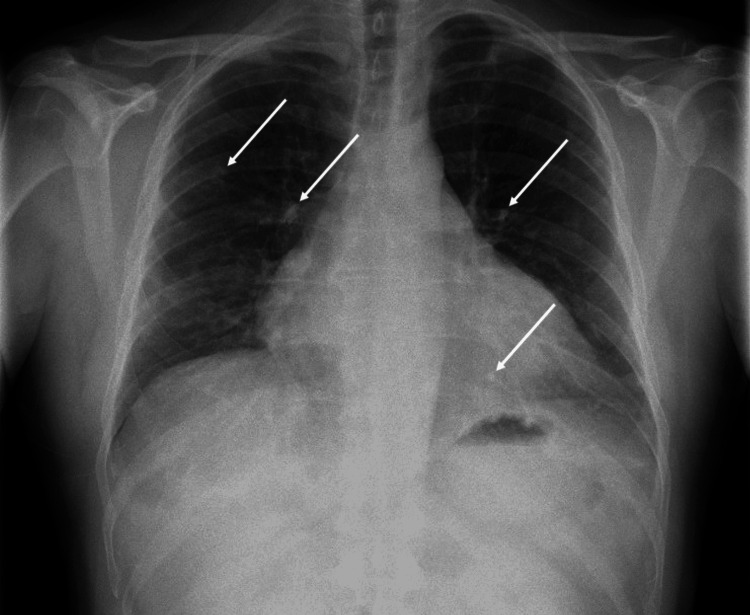
Admission chest X-ray identifying multifocal nodular opacities in both lung fields, stable cardiomegaly, and no pleural effusion

**Figure 2:**
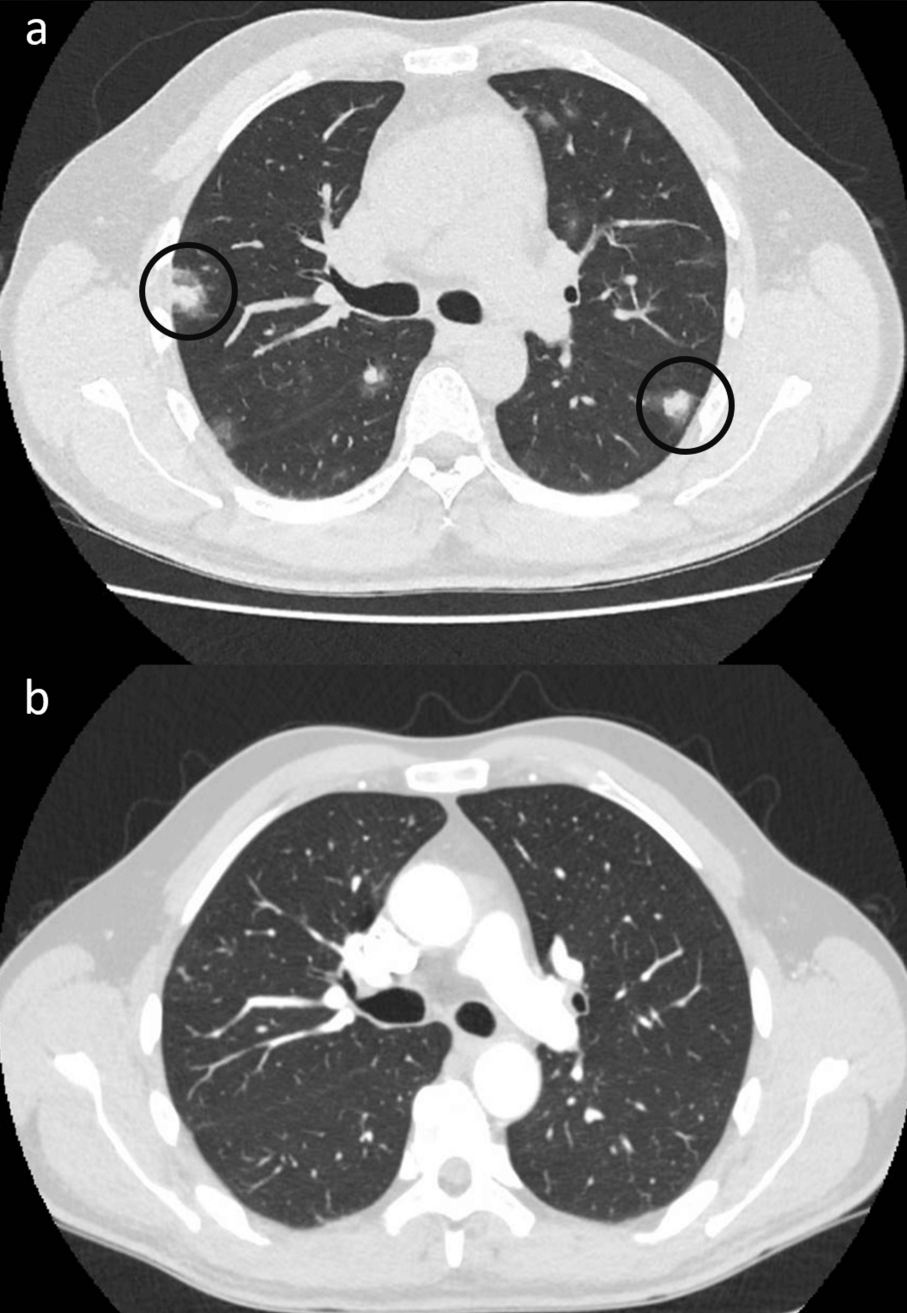
Computed tomography (CT) comparison of lung metastases (identified by circles) before systemic therapy (a) and on restaging imaging post-cycle six chemotherapy (b)

Routine blood work, including tumor markers, provided limited diagnostic yield (Tables 1, 2). Tuberculosis cultures were negative. Chest X-ray noted new multifocal nodular opacities in both lung fields, stable cardiomegaly, and no pleural effusion (Figure 1). CT thorax, abdomen, and pelvis (CT TAP) identified interval development of mediastinal calcified nodular lesions with mass effect on cardiac chambers, multifocal bilateral parenchymal nodular opacities with ground-glass halo suggestive of hemorrhage (Figure 2a), and diffusely abnormal pericardium with areas of calcification and nodularity. Lesions were also identified in the liver, thoracolumbar spine, left hemipelvis, and left second rib.

**Table 1:** Routine laboratory investigations

Investigation	Result	Reference Ranges
Full blood count (FBC), renal/liver/bone profiles	Normal range	-
Lactate dehydrogenase (LDH)	554	220-450 units per liter (U/L)
C-reactive protein (CRP)	9.9	0-5.0 milligrams per liter (mg/L)
D-dimer	6.66	0-0.50 mg/L
Autoinflammatory screen	Negative	-
Autoimmune screen	Antinuclear antibody (ANA) weak positive speckled	-
Infection screen	Hepatitis A immunoglobulin G (IgG) positive	-

**Table 2:** Tumor markers and further laboratory work

Investigation	Result	Reference Ranges
Prostate-specific antigen (PSA)	0.49	0-4.0 micrograms per liter (µg/L)
Carcinoembryonic antigen (CEA)	1.7	0-5.0 µg/L
Carbohydrate antigen (CA) 19-9	10	0-37.0 kilo units per liter (kU/L)
Alpha-fetoprotein (AFP)	5.3	0.90-8.80 µg/L
Beta-2 microglobulin	1.53	1.19-2.42 mg/L
Haptoglobin	3.61	0.40-1.60 grams per liter (g/L)

**Figure 3:**
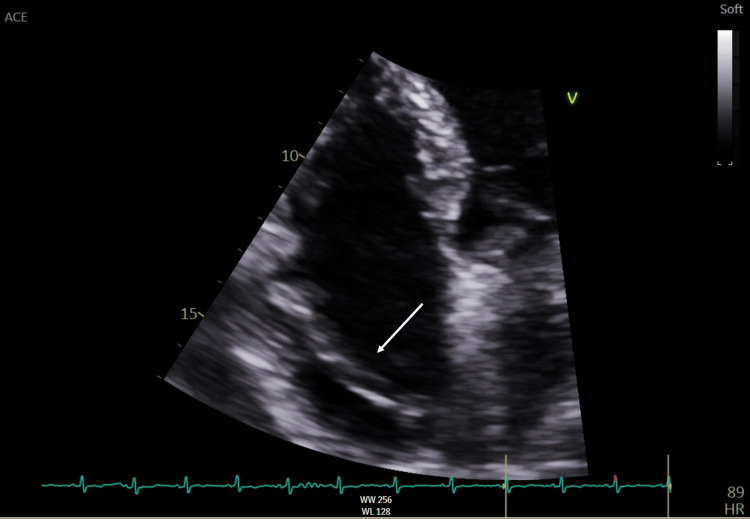
Transthoracic echocardiogram (TTE) identifying a large, highly mobile linear structure in the right atrium (arrow)

A transthoracic echocardiogram (TTE) identified a large, highly mobile linear structure in the right atrium and a mild/moderate localized pericardial effusion measuring 1.4 cm adjacent to the right atrium (Figure 3). A small patent foramen ovale was visualized. Bronchoscopy with bronchoalveolar lavage confirmed atypical squamous cells with a background of squamous metaplasia and hemosiderin-laden macrophages. There were no confirmatory features of malignancy. The pulmonary lesions were not amenable to percutaneous biopsy.

**Figure 4:**
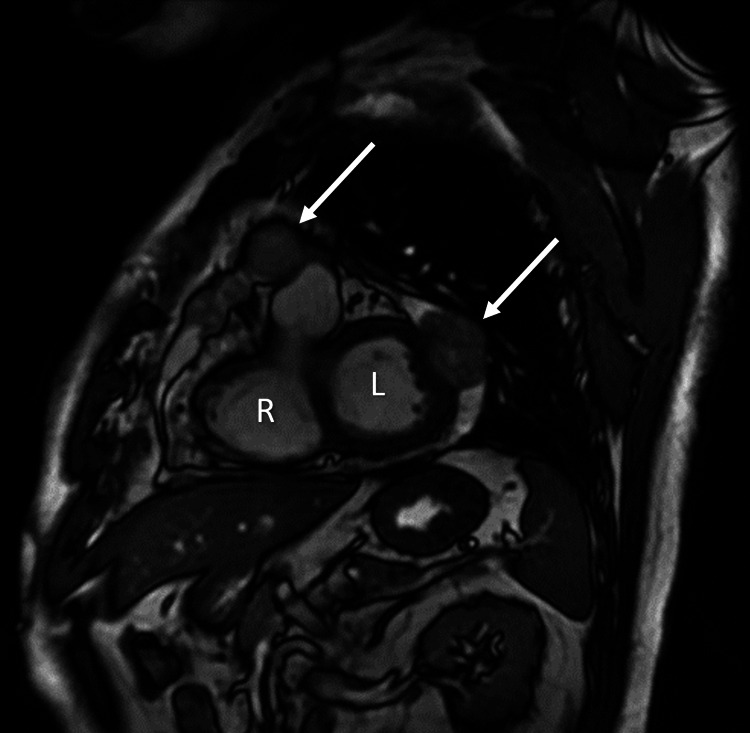
Cardiac magnetic resonance image (MRI) identifying mass-like right atrial and pericardial lesions (arrows), right ventricle (R), and left ventricle (L)

A cardiac magnetic resonance imaging (MRI) identified a 3.2 cm enhancing mass-like thickening of the right atrium and interatrial septum lying 3 cm inferior to the pulmonary artery. There were also enhancing necrotic-appearing pericardial nodules and an associated pericardial effusion (Figure 4).

Diagnosis

**Figure 5:**
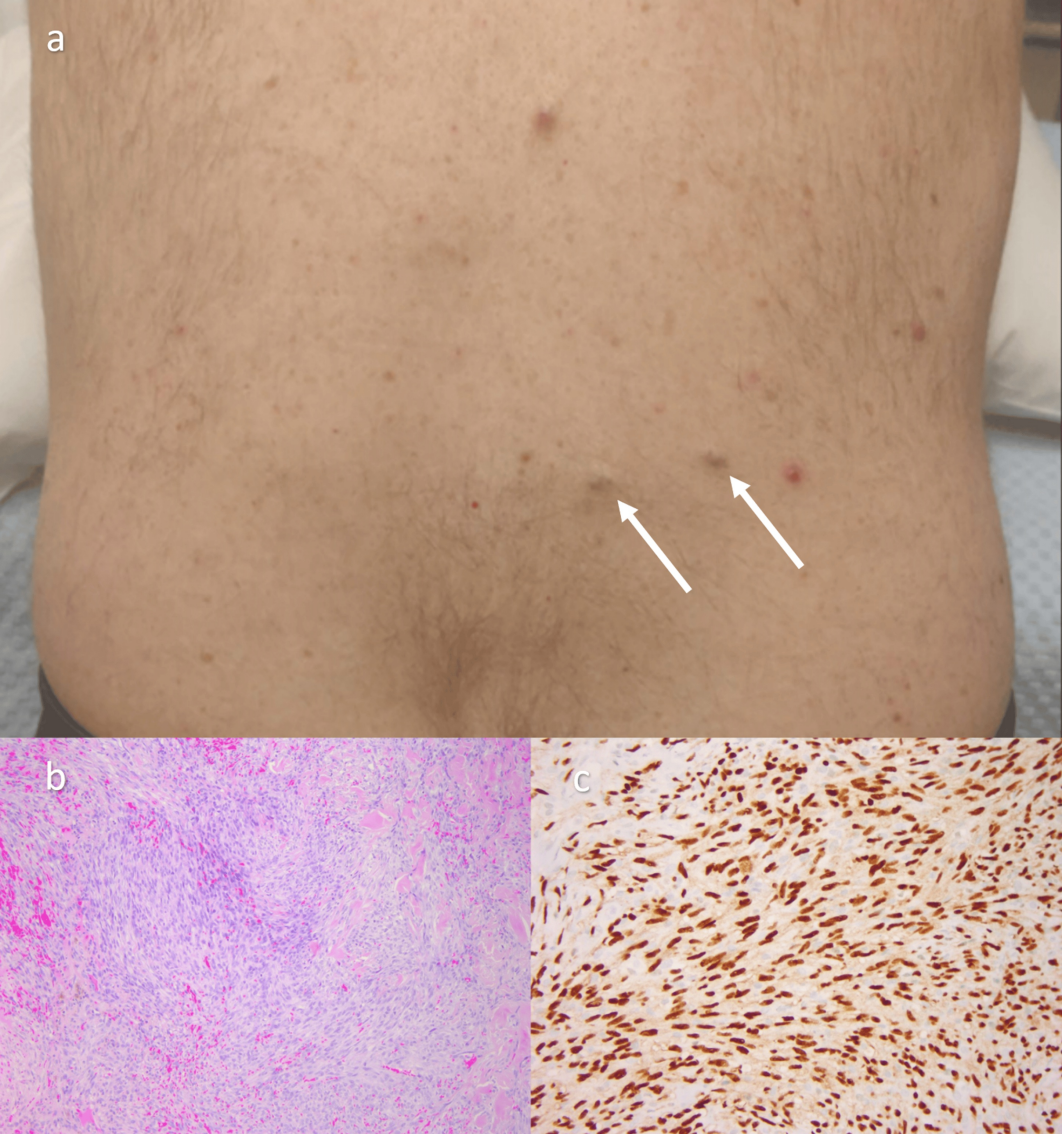
(a) Dermal metastases seen on skin examination (arrows), measuring 1 cm each. (b) Punch biopsy of the skin lesion. The lesion is very cellular and composed of a population of spindle cells. Extravasated red blood cells noted admixed with the tumor (5× H&E). (c) Strong nuclear staining of the lesion confirming the vascular origin of this tumor (20× ERG immunohistochemistry) H&E: hematoxylin and eosin; ERG: erythroblast transformation-specific regulated gene

During this admission, two new skin lesions appeared on the patient's lower back (Figure 5a). A dermatology review noted two firm, tender nodules measuring 1 cm each, and these were biopsied, avoiding other more invasive tissue sampling that had been planned. The histopathology report described nodules of an atypical spindle cell proliferation in the deep dermis with numerous mitoses and scattered red blood cells within the tumor. Cells were erythroblast transformation-specific regulated gene (*ERG*) and cluster of differentiation 31 (CD31) positive, confirming the vascular origin of the tumor and ruling out the differential diagnosis of a spindle cell/pleomorphic lipomatous tumor. Melanocytic markers S100 and Sry-related HMG-box 10 (*Sox10*) and keratin markers AE1 and AE3 were negative, ruling out melanocytic and epithelial origin malignancies, respectively. Immunohistochemistry for human herpesvirus-8 (HVV8) was negative, ruling out Kaposi's sarcoma. A diagnosis of stage IV metastatic cardiac angiosarcoma was reached by consensus following discussion at the soft tissue sarcoma multidisciplinary team meeting. A Kirsten rat sarcoma viral oncogene homolog (*KRAS*) V14I mutation (c.40G>A p.V14I @ 19% variant allele frequency (VAF)) was identified on genetic next-generation sequencing of the tumor cells (Figure 5).

Treatment and response

The patient commenced doxorubicin and ifosfamide chemotherapy with granulocyte colony-stimulating factor (GCSF) support and reported resolution of presenting symptoms following cycle one. Zoledronic acid was added in the context of bone metastases to reduce the risk of bone pain, pathologic fracture, and spinal cord compression. Clinical examination showed gross resolution of dermal metastases shortly after commencing chemotherapy. Restaging CT TAP after three cycles of chemotherapy showed a significant reduction in tumor burden. A second restaging CT TAP post-cycle six confirmed a further reduction in pulmonary metastases, pericardial thickening, and hepatic lesions. Osseous metastatic disease was radiologically stable. Chemotherapy was then stopped for a treatment break, and the patient was followed on surveillance.

**Figure 6:**
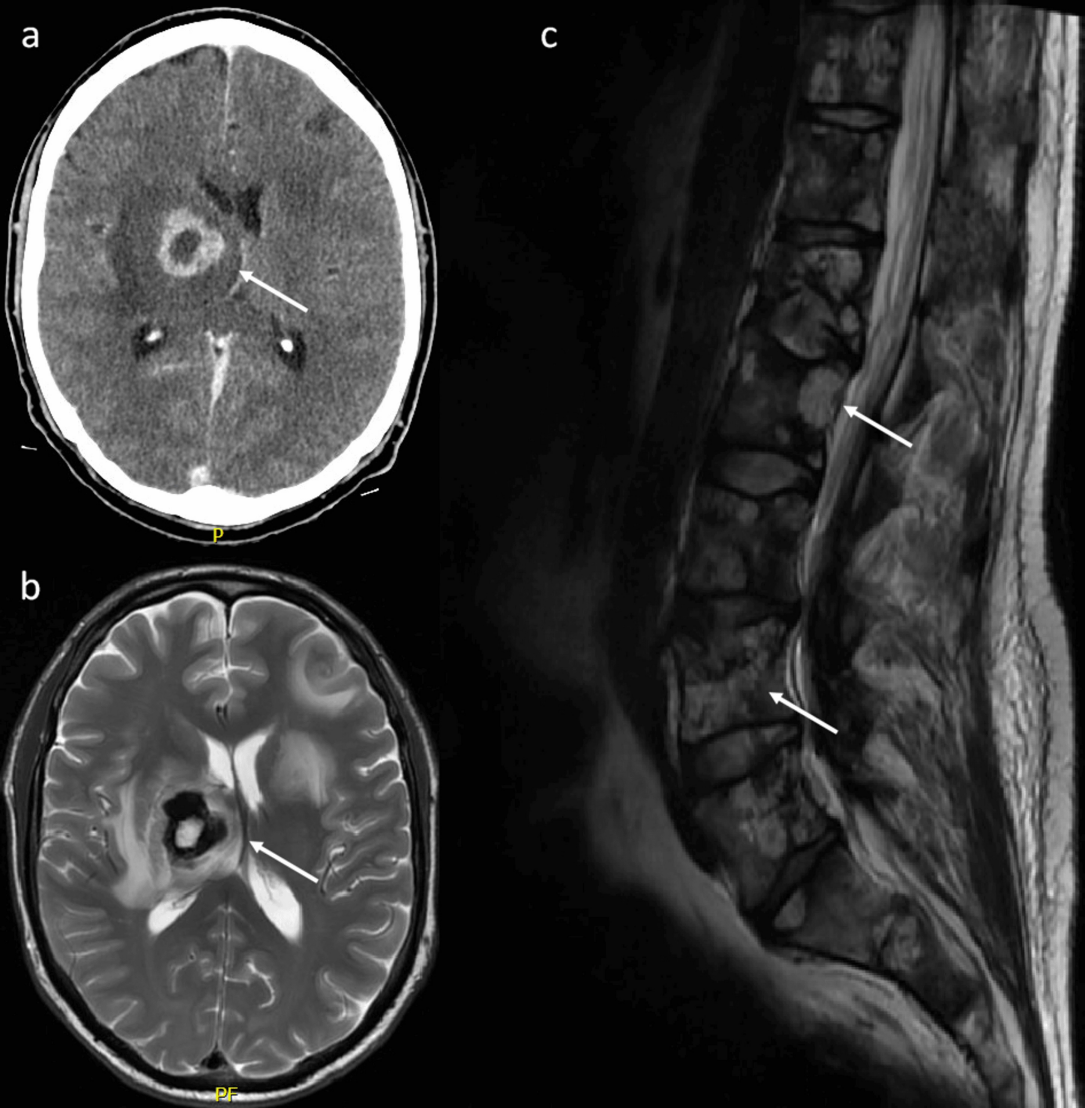
CT brain (a) and MRI brain (b) identifying intracranial metastases, and MRI lumbar spine (c) showing osseous metastatic disease, highlighted by arrows

**Figure 7:**
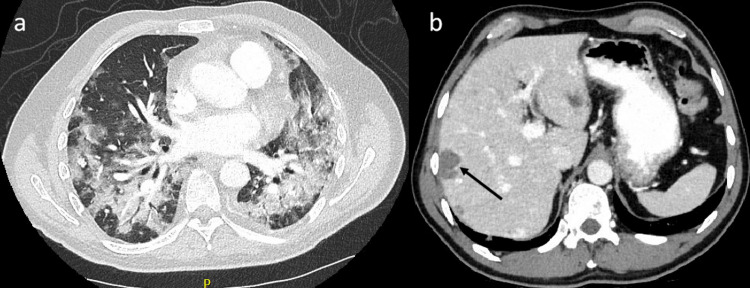
Repeat CT TAP images show progressive disease and superimposed pneumonia in the thorax (a), with increasing metastases in the liver (b) CT TAP: computed tomography of the thorax, abdomen, and pelvis

Three months after the completion of cycle six chemotherapy, the patient was admitted with left facial droop and left upper limb weakness. CT and MRI brain identified multiple new brain metastases (Figure 6). He commenced dexamethasone and received a course of whole-brain radiotherapy. CT TAP showed progressing lung, liver, and bone metastases. Weekly paclitaxel was initiated following the completion of radiotherapy. The patient experienced anemia and thrombocytopenia secondary to bone marrow suppression. A subsequent admission with acute back pain and increasing dyspnea identified an L1 vertebral pathologic fracture. MRI of the lumbar spine showed extensive osseous metastatic disease (Figure 6). CT TAP noted significant disease progression in the thorax and liver, with superimposed pneumonia (Figure 7). The patient was discharged on home oxygen, having discontinued paclitaxel, and was commenced on end-of-life care with palliative care team input. He died two days later, 10 months after diagnosis.

## Discussion

This case identifies an extremely rare case of metastatic cardiac angiosarcoma. Patients can present with myriad symptoms depending on organ involvement. Early metastases are a feature of angiosarcoma, which often spreads to the lung, liver, and bone. Initial symptoms are frequently vague, including dyspnea, fatigue, weight loss, atrial tachycardia, hypotension, and syncope. Cardiac tamponade can also develop [4]. The nonspecific nature of these symptoms provides limited diagnostic insight and leads to a broad differential diagnosis. Our patient's presentation included chest pain, proximal myalgia, pericarditis, cardiac tamponade, and hemoptysis as presenting features.

Diagnostic imaging findings are nonspecific, especially in the setting of diffuse metastases. Case reports in the literature note the halo sign on CT thorax as a feature of lung metastases in angiosarcoma [5]. The halo sign describes a ground-glass opacity surrounding a parenchymal lung mass and is reflective of alveolar hemorrhage and surrounding pulmonary infarction. It has a broad differential, including infections such as invasive pulmonary aspergillosis, sarcoidosis, primary lung neoplasms, and lung metastases [6]. Negative infectious, autoimmune, and auto-inflammatory screens narrowed the list of possible etiologies in this case. Approximately 80% of cardiac angiosarcomas originate at the right atrioventricular groove [4]. Other masses found in this location include atrial myxoma, thrombus, and cardiac metastases from another primary malignancy. A transthoracic echocardiogram, in our case, identified a mass at the right atrium and interatrial septum, providing further rationale for determining this patient to have a right atrial primary tumor.

Tissue diagnosis in cardiac angiosarcoma is often challenging to achieve, even in the setting of widespread metastases. Pericardial and pleural fluid sampling is often nondiagnostic [5,7]. Here, the diagnosis was reached following a punch biopsy of a cutaneous lesion identified on full skin examination. This avoided higher risk and more invasive sampling for tissue diagnosis, which had been planned. Diagnosis of primary cardiac angiosarcoma following a biopsy of a cutaneous metastasis is exceptionally rare. Two cases of diagnosis via skin biopsy were identified in the literature [8,9]. Both cases described angiosarcomas that originated in the left atrium, whereas the present case originated in the right atrium.

Histopathological examination and immunohistochemistry provided key diagnostic insight in this case. Histological mimickers of angiosarcoma include Kaposi sarcoma, spindle cell hemangioma, epithelial carcinoma, and melanoma [3]. CD31 positive staining of the skin lesion biopsy confirmed the vascular lumen origin of the dermal metastases. Positive *ERG* immunohistochemistry is highly specific for both benign and malignant vascular tumors, including angiosarcomas and Kaposi sarcomas. The negative HVV8 test ruled out Kaposi sarcoma, aiding the correct diagnosis.

Discussion at the soft tissue sarcoma multidisciplinary team meeting determined that the primary tumor in this case originated in the right atrium. This decision was reached on consideration of the clinical and radiological features, as well as the histopathological and immunohistochemical assessment of the skin biopsy.

Metastatic angiosarcoma has a median overall survival of 3-10 months. A consensus on treatment strategy has not been established to date, partly due to its rarity. Surgical resection can be considered in localized disease. Systemic chemotherapy options in the metastatic setting include doxorubicin and weekly paclitaxel. Targeted therapies that have undergone investigation in this setting include vascular endothelial growth factor (VEGF) inhibitors such as bevacizumab and tyrosine kinase inhibitors such as apatinib [3]. Doxorubicin/ifosfamide chemotherapy induced a good response for this patient, albeit short-lived. There was some concern about using an anthracycline-based regimen given the potential cardiotoxicity of these agents and the underlying cardiac malignancy, which was impairing cardiac function. However, on discussion with the patient's cardiologist, it was felt he had good underlying cardiac reserve given his lack of any pre-existing atherosclerotic or other cardiac issues, so the oncologic benefits of anthracycline-based treatment were adjudged to outweigh the risks.

Molecular analysis of this tumor revealed an activating *KRAS* mutation as the oncogenic driver mutation for this tumor. The *KRAS* V14I mutation identified in our case has been described in a previously reported case of skin angiosarcoma [10]. Genetic alterations affecting the mitogen-activated protein kinase (MAPK) pathway have been noted in genomic analyses of angiosarcomas. In one study of 34 angiosarcomas, MAPK pathway alterations were identified in 53% of cases, including one tumor that was positive for a *KRAS* G12D mutation [11]. The development of appropriate *KRAS*-targeting drugs may be a therapeutic avenue worth exploring for these patients through appropriately designed clinical trials. Early-phase clinical trials investigating *KRAS* inhibitors in participants with advanced or metastatic solid organ malignancies include the *KRAS* G12C inhibitor MK1084 [12] and BGB-53038, a pan-*KRAS* inhibitor [13].

## Conclusions

This case provides insight into the detection and management of a rare malignancy, which was diagnosed on punch biopsy of a cutaneous metastasis. This presentation of a rare cancer had a broad differential, requiring a considered physical examination and tissue sampling to reach the diagnosis. The development of cutaneous metastases in a case of right atrial cardiac angiosarcoma is noteworthy in the published literature. Doxorubicin-based chemotherapy was used with some success despite the risk of cardiotoxicity in the setting of a primary cardiac tumor. CT imaging allowed for non-invasive monitoring of treatment response. Research on previously "undruggable" *KRAS* mutations may offer hope for patients in the future via clinical trial enrollment.
